# Thromboxane biosynthesis and future events in diabetes: the ASCEND trial

**DOI:** 10.1093/eurheartj/ehad868

**Published:** 2024-02-22

**Authors:** Giovanna Petrucci, Georgina A Buck, Bianca Rocca, Sarah Parish, Colin Baigent, Duaa Hatem, Marion Mafham, Aida Habib, Louise Bowman, Jane Armitage, Carlo Patrono

**Affiliations:** Section of Pharmacology, Catholic University School of Medicine, Largo F. Vito 1, Rome 00168, Italy; Clinical Trial Service Unit and Epidemiological Studies Unit, Nuffield Department of Population Health, University of Oxford, Richard Doll Building, Old Road Campus, Oxford OX3 7LF, UK; Section of Pharmacology, Catholic University School of Medicine, Largo F. Vito 1, Rome 00168, Italy; Clinical Trial Service Unit and Epidemiological Studies Unit, Nuffield Department of Population Health, University of Oxford, Richard Doll Building, Old Road Campus, Oxford OX3 7LF, UK; Medical Research Council Population Health Research Unit, Nuffield Department of Population Health, University of Oxford, Richard Doll Building, Old Road Campus, Oxford OX3 7LF, UK; Clinical Trial Service Unit and Epidemiological Studies Unit, Nuffield Department of Population Health, University of Oxford, Richard Doll Building, Old Road Campus, Oxford OX3 7LF, UK; Medical Research Council Population Health Research Unit, Nuffield Department of Population Health, University of Oxford, Richard Doll Building, Old Road Campus, Oxford OX3 7LF, UK; Section of Pharmacology, Catholic University School of Medicine, Largo F. Vito 1, Rome 00168, Italy; Clinical Trial Service Unit and Epidemiological Studies Unit, Nuffield Department of Population Health, University of Oxford, Richard Doll Building, Old Road Campus, Oxford OX3 7LF, UK; Department of Basic Medical Sciences, College of Medicine, QU Health, Qatar University, Doha, Qatar; Clinical Trial Service Unit and Epidemiological Studies Unit, Nuffield Department of Population Health, University of Oxford, Richard Doll Building, Old Road Campus, Oxford OX3 7LF, UK; Medical Research Council Population Health Research Unit, Nuffield Department of Population Health, University of Oxford, Richard Doll Building, Old Road Campus, Oxford OX3 7LF, UK; Clinical Trial Service Unit and Epidemiological Studies Unit, Nuffield Department of Population Health, University of Oxford, Richard Doll Building, Old Road Campus, Oxford OX3 7LF, UK; Medical Research Council Population Health Research Unit, Nuffield Department of Population Health, University of Oxford, Richard Doll Building, Old Road Campus, Oxford OX3 7LF, UK; Section of Pharmacology, Catholic University School of Medicine, Largo F. Vito 1, Rome 00168, Italy

**Keywords:** Randomized placebo-controlled trial, Daily low-dose aspirin, Urinary 11-dehydro-thromboxane B_2_, Platelet activation, Diabetes

## Abstract

**Background and Aims:**

Thromboxane (TX) A_2_, released by activated platelets, plays an important role in atherothrombosis. Urinary 11-dehydro-TXB_2_ (U-TXM), a stable metabolite reflecting the whole-body TXA_2_ biosynthesis, is reduced by ∼70% by daily low-dose aspirin. The U-TXM represents a non-invasive biomarker of *in vivo* platelet activation and is enhanced in patients with diabetes. This study assessed whether U-TXM is associated with the risk of future serious vascular events or revascularizations (SVE-R), major bleeding, or cancer in patients with diabetes.

**Methods:**

The U-TXM was measured pre-randomization to aspirin or placebo in 5948 people with type 1 or 2 diabetes and no cardiovascular disease, in the ASCEND trial. Associations between log U-TXM and SVE-R (*n* = 618), major bleed (*n* = 206), and cancer (*n* = 700) during 6.6 years of follow-up were investigated by Cox regression; comparisons of these associations with the effects of randomization to aspirin were made.

**Results:**

Higher U-TXM was associated with older age, female sex, current smoking, type 2 diabetes, higher body size, urinary albumin/creatinine ratio of ≥3 mg/mmol, and higher estimated glomerular filtration rate. After adjustment for these, U-TXM was marginally statistically significantly associated with SVE-R and major bleed but not cancer [hazard ratios per 1 SD higher log U-TXM (95% confidence interval): 1.09 (1.00–1.18), 1.16 (1.01–1.34), and 1.06 (0.98–1.14)]. The hazard ratio was similar to that implied by the clinical effects of randomization to aspirin for SVE-R but not for major bleed.

**Conclusions:**

The U-TXM was log-linearly independently associated with SVE-R in diabetes. This is consistent with the involvement of platelet TXA_2_ in diabetic atherothrombosis.

## Structured Graphical Abstract

**Structured Graphical Abstract:**
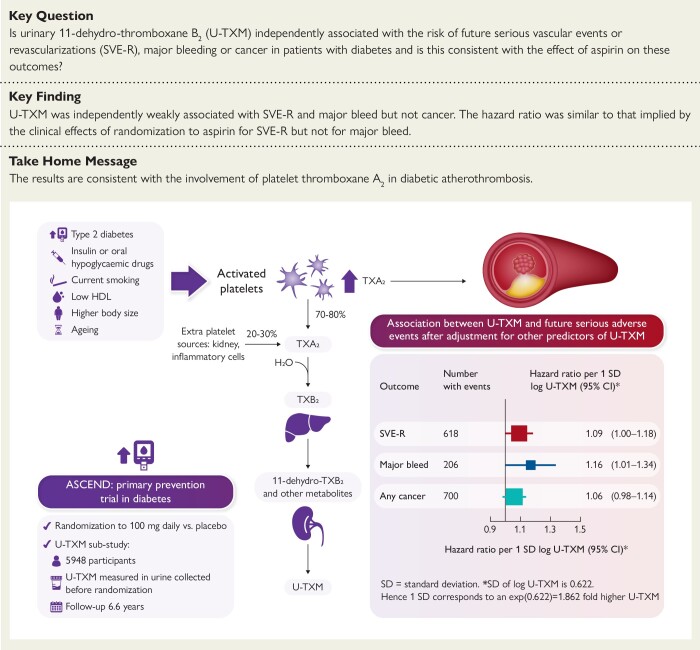
Urinary 11-dehydro-thromboxane B_2_ is associated with the risk of future serious vascular events or revascularizations in the urinary 11-dehydro-thromboxane B_2_ sub-study of the ASCEND trial in diabetes. CI, confidence interval; HDL, high-density lipoprotein; SD, standard deviation; SVE-R, serious vascular events or revascularizations; TX, thromboxane; U-TXM, urinary 11-dehydro-TXB_2_.

**See the editorial comment for this article ‘Urinary thromboxane and risk of cardiovascular events: role of aspirin’, by J.W. Eikelboom and P.C. Kruger, https://doi.org/10.1093/eurheartj/ehae003.**

## Introduction

The antiplatelet effects of cyclooxygenase (COX)-1 inhibition, such as by low-dose aspirin, are an important component of cardiovascular disease prevention. Thromboxane (TX) A_2_, a short-lived lipid mediator released by activated platelets,^1^ promotes platelet aggregation and vasoconstriction and is involved in several pathophysiologic processes including primary haemostasis, atherosclerosis progression and thrombosis, inflammation, and cancer.^2–4^ The enzymatic metabolism of TXA_2_ and its stable hydrolysis product, TXB_2_, leads to a stable end-product, 11-dehydro-TXB_2_ (TXM), which is measurable in urine and reflects the whole-body rate of TXA_2_ biosynthesis.^5^ Chronic treatment with low-dose aspirin, by permanently inactivating platelet COX-1, reduces TXM excretion by 70%–80%.^6,7^ Hence, urinary TXM (U-TXM) excretion is a non-invasive index of *in vivo* platelet activation, and its measurement has been instrumental in identifying clinical conditions in which low-dose aspirin therapy is of benefit.^2^ Increased platelet activation, as reflected by elevated U-TXM excretion, has been reported in association with major cardiovascular risk factors that accelerate atherogenesis, including diabetes mellitus.^8^ In addition, several lines of evidence support a role of platelet activation in some cancers, particularly colorectal cancer, leading to the hypothesis that platelet COX-1 inhibition may contribute to the postulated chemopreventive effect of low-dose aspirin.^9,10^

The ASCEND (A Study of Cardiovascular Events in Diabetes) randomized trial assessed the efficacy and safety of 100 mg daily enteric-coated aspirin, compared with placebo for the primary prevention of vascular events amongst 15 480 people who had type 1 or type 2 diabetes but no evidence of cardiovascular disease at trial entry.^11^ It found aspirin reduced the risk of serious vascular events or revascularizations (SVE-R) but increased the risk of a major bleed,^12^ consistent with the involvement of platelet TXA_2_ production in both atherothrombosis and primary haemostasis in this setting.^13^ There was no evidence of an effect on gastrointestinal tract cancer or cancer at any other site during the scheduled follow-up period.^12^ In a sub-study amongst 152 participants, randomization to aspirin was also associated with a 71% reduction in U-TXM.^7^

The aims of the present ASCEND sub-study were as follows: (i) to investigate the association between baseline U-TXM and future SVE-R, major bleeds, and incident cancers independent of other risk factors and treatment and (ii) to compare these observational associations with the expected effects based on the randomized comparison and thereby investigate whether the effect of aspirin on outcomes was consistent with mediation via its effect on platelet TXA_2_ biosynthesis.

## Methods

The methods, baseline characteristics, and main results of ASCEND (ISRCTN.com: ISRCTN60635500; ClinicalTrials.gov: NCT00135226) have been described elsewhere.^11,12,14^ The trial was approved by the North West Multicentre Research Ethics Committee and all participants provided written informed consent. In brief, people with diabetes (type 1 or 2) were identified from centrally held diabetes registers (e.g. for retinopathy screening) and from UK general practices and invited by mail to take part in the trial. A total of 423 403 were invited, 26 462 entered a 2-month pre-randomization run-in phase, and 15 480 were randomized. During the pre-randomization run-in, participants were asked to stop any previous aspirin therapy and were supplied with placebo aspirin and placebo n-3 fatty acids to be taken once daily. Participants were sent a blood and urine testing kit to take to their local general practice and asked to provide samples that were then mailed to the ASCEND central laboratory. The sampling was optional and participants could still be randomized even if samples had not been received. Urine samples were collected into containers without preservative and mailed at room temperature. Samples arriving in the central laboratory were aliquoted and frozen at −80°C or below before being shipped on dry ice to the Pharmacology Department of the Catholic University in Rome. A preliminary study was conducted to assess the stability of TXM under the collection and mailing conditions of urine samples collected from ASCEND participants.^15^

### Materials and laboratory methods

Urine samples of 1 mL were assayed when available. If the available volume was <1 mL, then 0.5 mL was used for chromatographic extraction. The detailed methodology for the U-TXM measurement,^15^ the protocol for the preliminary assessment of U-TXM stability, and a list of consumables and reagents are provided in the Supplementary data online. Final U-TXM values were divided by urine creatinine to correct for urine concentration and were expressed as picograms per milligram of creatinine. Only U-TXM at baseline was assessed in this sub-study. As baseline samples were taken during the 2-month run-in period when participants had ceased previous aspirin and considering that U-TXM levels recover within 72 h of aspirin withdrawal,^16^ baseline U-TXM is considered to be off aspirin. Further urine samples were collected a mean of 2 years after randomization in a random sample of around 10% participants and a previously reported sub-study of ASCEND, independent of the present study, assessed U-TXM at baseline and follow-up in a random sample of 152 participants selected from those who had urine samples available at baseline and follow-up that were received by the laboratory 1–2 days after the sample was taken.^7^ However, U-TXM was not assessed in the remaining follow-up urine samples, and there were insufficient numbers to investigate associations between follow-up U-TXM and outcome.

### Follow-up and outcomes

After randomization, information was sought about all serious adverse events (including potential trial outcomes) through 6-monthly follow-up questionnaires. Three of the trial primary and secondary outcomes (all of which were adjudicated) are considered in this sub-study: first SVE-R, defined as a composite of non-fatal myocardial infarction, non-fatal stroke (excluding confirmed intracranial haemorrhage), transient ischaemic attack, any arterial revascularization procedure, or death from any vascular cause (excluding confirmed intracranial haemorrhage); first occurrence of any major bleeding event, defined as a composite of any confirmed intracranial haemorrhage, sight-threatening bleeding event in the eye, gastrointestinal bleeding, or any other serious bleeding (i.e. a bleeding event that resulted in hospitalization or transfusion or that was fatal); and first occurrence of any cancer. Constituent events of the main outcomes were also investigated, as was a composite of all non-vascular, non-cancer outcomes classified by the Medical Dictionary for Regulatory Activities (MedDRA), which was investigated as a negative control, i.e. with no association with U-TXM hypothesized.

### Statistical analyses

**Figure 1:**
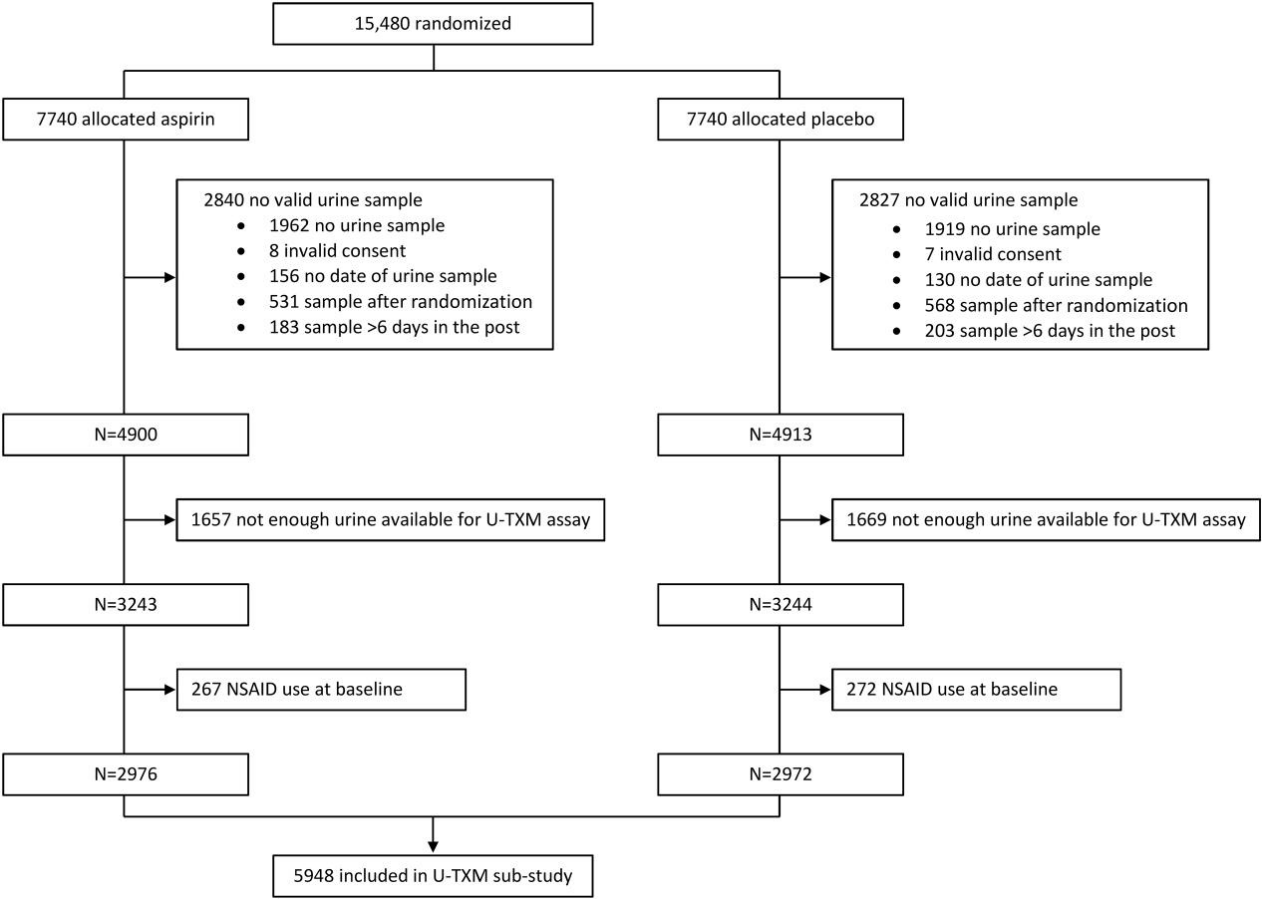
Flow diagram of eligibility of urine samples for urinary 11-dehydro-thromboxane B_2_ sub-study. NSAID, non-steroidal anti-inflammatory drug; U-TXM, urinary 11-dehydro-thromboxane B_2_

Analyses excluded participants who did not give consent to analyse samples or those whose baseline sample was collected after randomization (*Figure 1*). Urine samples that took more than 6 days to arrive at the central ASCEND laboratory were excluded due to the potential instability of U-TXM at ambient temperature for this length of time.^15^ Participants using non-steroidal anti-inflammatory drugs (NSAIDs) at baseline were excluded because of the known inhibitory effects of NSAIDs on TXA_2_ biosynthesis and U-TXM excretion.^17^ Urinary TXM appeared log-normally distributed. Analyses of outcomes were by quartiles and per SD (= 0.622) of continuous log U-TXM (i.e. 1 SD corresponds to an exp(0.622) = 1.86-fold higher U-TXM). For the purpose of identifying potential confounders, linear regression models were fitted using stepwise selection to determine predictors of log U-TXM, with the basic factors [sex, 5-year age groups, and urine volume assayed (0.5 vs. 1 mL)] and randomized treatment allocation (aspirin vs. placebo) forced into the model and further main effects and pairwise interactions (reducing smoking to current/not and age to three 10 year groups in the interaction terms) added to the model if they had a *P*-value of <.05 but removed if the *P*-value was >.05. To enable all participants with eligible U-TXM to be included in the stepwise selection model, those with missing baseline categorical data were assigned to a separate category if 10 or more cases were missing or to the most common category where numbers missing were smaller than this. As the number of missing values was low, less than 1% for all baseline measures except body mass index (3.3%), systolic and diastolic blood pressure (3.6%), and duration of diabetes (5.5%), missing baseline continuous data were simply assigned the sex-specific mean value, with a data availability indicator variable included as a potential factor.

Cox regression was used to investigate the association between log U-TXM and time from randomization to the first occurrence of each type of event of interest during the scheduled follow-up period, with complete follow-up information available for over 99% of participants.^12^ Those who withdrew or were lost to follow-up were censored on the date of last contact before this. Based on other studies^13,18–20^ and in the previous sub-study of 152 participants in the ASCEND trial,^7^ U-TXM would be expected to be about 70% lower on aspirin. However, any association between U-TXM and outcomes would be expected to pertain to populations on and off aspirin.^21^ Therefore, to maximize the data included, the associations between log U-TXM data and outcomes were analysed using data from both the aspirin and placebo-randomized arms with treatment allocation included as a factor in all analyses.

The effects of adjustment for the baseline predictors of log U-TXM on the associations of log U-TXM with outcomes were assessed. Following the rule of thumb that there should be at least 10 events per parameter for robust modelling,^22,23^ and with approximately 200 events in the least frequent outcome (major bleed), a number of parameters in adjusted analyses were restricted to 20 (19 adjustment terms from the stepwise selection model plus a U-TXM term). The main analyses were therefore adjusted for basic factors and the most significant baseline predictors of log U-TXM. Hazard ratios (HRs) across U-TXM quartiles within each treatment arm were presented as floating absolute risks relative to the lowest quartile in the placebo arm (whereby each category is assigned a ‘floated’ standard error to describe the uncertainty in the risk independent of other categories, thus avoiding comparisons of categories to an arbitrary reference group).^24^ An HR per 1 SD higher log U-TXM, constrained to be the same in the placebo and aspirin arms, was estimated from a model including randomized allocation to aspirin as an explanatory factor but with no interaction term between treatment allocation and log U-TXM. The likelihood ratio test was used to estimate *P*-values for associations of continuous log U-TXM with outcomes. Hazard ratios per 1 SD higher log U-TXM are presented with 95% confidence intervals (CIs). Sensitivity analyses were undertaken to investigate for any heterogeneity in the effects between the aspirin and placebo arms.

To assess whether the above observational analyses were consistent with the hypothesis that the effect of aspirin on outcomes is mediated by the inhibitory effects of aspirin on TXA_2_ biosynthesis, we considered the effects of randomization to aspirin on outcomes and on U-TXM. In a randomized comparison in the previous sub-study of 152 randomly selected ASCEND participants, a single measure of U-TXM measured a mean of 2 years after randomization was 71% (95% CI 64%–76%, *P* < .001) lower in those allocated low-dose aspirin vs. those allocated placebo, based on the 150 participants with quantified U-TXM at baseline.^7^ In this present study, based on 71% = 100 × (1 − exp[−1.222]), this was taken to correspond to a 1.222/0.622 = 1.96 SD lower log U-TXM with allocation to aspirin. Using this information, HRs for the randomized effect of aspirin on outcomes were expressed as HRs per 1 SD higher log U-TXM, for comparison with the observational results. To illustrate graphically the comparative outcome risks by estimated on-treatment U-TXM levels, HRs in the placebo group quartiles were plotted at geometric mean baseline levels of U-TXM (i.e. assuming on-placebo U-TXM levels are similar to baseline levels) and the HR in the aspirin arm relative to the placebo arm was plotted at 392 pg/mg creatinine, 71% lower than the geometric mean baseline U-TXM (1330 pg/mg) (i.e. assuming a similar reduction in U-TXM with aspirin to that seen in the previous ASCEND sub-study).^7^

Statistical analyses and plotting used SAS version 9.4 (SAS Institute Inc., Cary, NC, USA) and R version 3.6.2 in RStudio version 1.2.5033.

## Results

Amongst the 15 480 participants randomized in ASCEND, 5948 participants (2976 allocated aspirin and 2972 placebo) had a valid baseline and pre-randomization U-TXM assay and were included in the analyses (*Figure 1*). The baseline characteristics and geometric mean U-TXM are shown in *Table 1*. Those with eligible baseline urine samples and U-TXM assays were older, had somewhat higher baseline vascular risk score, were more likely to have type 2 diabetes, and were less likely to be current smokers than the ASCEND population overall. The participants in this present study had similar characteristics to the 152 included in the previous ASCEND sub-study of U-TXM^7^ (given the chance differences to be expected in a small subsample; see Supplementary data online, *Table S1*).

**Table 1:** Baseline characteristics by availability of a valid urine sample and urinary 11-dehydro thromboxane B2 measure

Characteristic	All participants N (%)	Valid baseline urine sample available^a^		
		All N (%)	U-TXM measure available	
			N (%)	U-TXM geometric mean (95% CI), pg/mg creatinine
Overall	14 147	8975	5948	1330 (1309–1351)
Age at randomization (years)				
<60	5062 (35.8)	2768 (30.8)	1558 (26.2)	1314 (1274–1356)
≥60, <70	5690 (40.2)	3719 (41.4)	2615 (44.0)	1298 (1268–1329)
≥70	3395 (24.0)	2488 (27.7)	1775 (29.8)	1394 (1354–1435)
Sex				
Male	8950 (63.3)	5627 (62.7)	3802 (63.9)	1290 (1266–1316)
Female	5197 (36.7)	3348 (37.3)	2146 (36.1)	1404 (1366–1442)
Randomized allocation				
Placebo	7077 (50.0)	4500 (50.1)	2972 (50.0)	1317 (1288–1346)
Aspirin	7070 (50.0)	4475 (49.9)	2976 (50.0)	1344 (1314–1374)
Body mass index (kg/m^2^)				
<25	2132 (15.1)	1358 (15.1)	863 (14.5)	1325 (1273–1380)
≥25, <30	5136 (36.3)	3275 (36.5)	2177 (36.6)	1275 (1242–1308)
≥30	6440 (45.5)	4046 (45.1)	2709 (45.5)	1379 (1347–1413)
Unknown	439 (3.1)	296 (3.3)	199 (3.3)	1318 (1206–1441)
Type of diabetes				
Type 1	858 (6.1)	486 (5.4)	214 (3.6)	1052 (973–1138)
Type 2	13 289 (93.9)	8489 (94.6)	5734 (96.4)	1342 (1321–1364)
Diabetes management in type 2 diabetes				
Diet	2269 (17.1)	1485 (17.5)	1021 (17.8)	1234 (1186–1284)
Oral hypoglycaemic	8244 (62.0)	5231 (61.6)	3673 (64.1)	1369 (1342–1396)
Insulin	2776 (20.9)	1773 (20.9)	1040 (18.1)	1358 (1307–1411)
Smoking status				
Current	1144 (8.1)	658 (7.3)	396 (6.7)	1844 (1733–1962)
Former	6443 (45.5)	4190 (46.7)	2854 (48.0)	1333 (1303–1364)
Never	6399 (45.2)	4011 (44.7)	2619 (44.0)	1262 (1234–1292)
Unknown	161 (1.1)	116 (1.3)	79 (1.3)	1350 (1167–1562)
Baseline 5-year risk of serious vascular event				
Low (<5%)	5678 (40.1)	3445 (38.4)	2122 (35.7)	1281 (1247–1315)
Moderate (≥5%, <10%)	5992 (42.4)	3823 (42.6)	2642 (44.4)	1318 (1288–1348)
High (≥10%)	2477 (17.5)	1707 (19.0)	1184 (19.9)	1455 (1402–1509)
High-density lipoprotein cholesterol (mmol/L)				
<1.0	2258 (16.0)	1912 (21.3)	1228 (20.6)	1438 (1387–1490)
≥1.0, <1.5	5927 (41.9)	5031 (56.1)	3439 (57.8)	1329 (1302–1357)
≥1.5	2338 (16.5)	1927 (21.5)	1233 (20.7)	1229 (1186–1273)
No result available	3624 (25.6)	105 (1.2)	48 (0.8)	1500 (1285–1751)
Estimated glomerular filtration rate (mL/min/1.73 m^2^)				
<60	1310 (9.3)	1158 (12.9)	762 (12.8)	1356 (1291–1425)
≥60, <90	4171 (29.5)	3617 (40.3)	2382 (40.0)	1368 (1334–1403)
≥90	4837 (34.2)	4107 (45.8)	2756 (46.3)	1289 (1260–1318)
No result available	3829 (27.1)	93 (1.0)	48 (0.8)	1500 (1285–1751)
Urinary albumin–creatinine ratio (mg/mmol)				
<3	9192 (65.0)	7784 (86.7)	5135 (86.3)	1312 (1290–1334)
≥3	1408 (10.0)	1186 (13.2)	808 (13.6)	1454 (1390–1521)
No result available	3547 (25.1)	5 (0.1)	5 (0.1)	1526 (499–4663)

CI, confidence interval; N, number of people; U-TXM, urinary 11-dehydro-thromboxane B_2_.

^a^Urine samples were considered valid if the participant gave consent to analyse samples; sample was collected before randomization and arrived at the central ASCEND laboratory within 6 days. Excludes 1333 participants using non-steroidal anti-inflammatory drugs (NSAIDs) at baseline, 838 with a valid urine sample. Amongst those with a valid urine sample and U-TXM measured, geometric mean U-TXM was 912 pg/mg creatinine in NSAID users (*n* = 539) compared with 1330 pg/mg creatinine in non-users (*n* = 5948).

Urine extraction and analytical procedures were consistent over the entire study duration, as shown by the spiking experiments with exogenous TXM, whose recoveries were reproducible over time (see Supplementary data online, *Figures S1*–*S4*). The inter-assay precision of the enzyme-linked immunoassays was calculated by means of the coefficient of variation of the same internal standards and was 9.1% in a total of 1167 assays, which is considered as a high inter-assay precision.^25^

Statistically significant predictors of higher log U-TXM included female sex, older age, current smoking, type 2 diabetes, especially when treated with insulin or oral hypoglycaemic drugs, higher body mass index, urinary albumin/creatinine ratio of ≥3 mg/mmol, higher estimated glomerular filtration rate (calculated from blood cystatin C concentration using the Chronic Kidney Disease Epidemiology Collaboration formula^26^), no use of aspirin before screening, lower high-density lipoprotein cholesterol, and lower systolic blood pressure (*Table 2*). Amongst the 5948 participants included, 618 participants suffered a first SVE-R during a mean follow-up of 6.6 years, 206 had a major bleed, and 700 had cancer.

**Table 2:** Predictors of log urinary 11-dehydo thromboxane B2 from stepwise generalized linear model

Baseline factor	Estimate (SE)	*P*-value	Model degrees of freedom	Per cent of total sum of squares		Step^a^
				Factor	Model	
**Urine volume assayed 0.5 mL (vs. 1.0 mL)**	−0.18 (0.02)	2.5×10^−16^	1	1.12%	1.12%	0
**Female (vs. male)**	0.12 (0.02)	1.4 × 10^−06^	2	0.42%	1.55%	0
**Age group** <55	0.00	0.00045	7	0.33%	1.88%	0
≥55, <60	0.08 (0.03)					
≥60, <65	0.05 (0.03)					
≥65, <70	0.09 (0.03)					
≥70, <75	0.15 (0.03)					
≥75	0.24 (0.04)					
**Allocated aspirin (vs. placebo)**	0.02 (0.02)	0.15	8	0.03%	1.91%	0
**Smoker** Current	0.39 (0.03)	1.4 × 10^−32^	11	2.47%	4.38%	1
Former	0.06 (0.02)					
Unknown	0.07 (0.07)					
Never	0.00					
**High-density lipoprotein cholesterol (per mmol/L)**	−0.14 (0.03)	1.6 × 10^−14^	12	0.94%	5.33%	2
**Diabetes group** Type 2 diet only	0.05 (0.05)	3.7 × 10^−07^	14	0.47%	5.80%	3
Type 2 oral hypoglycaemic or insulin	0.13 (0.04)					
Type 1	0.00					
**Body mass index (per 5 kg/m^2^)**	0.04 (0.01)	2.4 × 10^−05^	15	0.28%	6.08%	4
**Urinary albumin-creatinine ratio ≥ 3 (vs. <3 mg/mmol)**	0.09 (0.02)	0.0014	16	0.16%	6.24%	5
**Estimated glomerular filtration rate (per 10mL/min/1.73 m^2^)**	0.01 (0.00)	0.0021	17	0.15%	6.39%	6
**Aspirin use before screening (vs. not)**	−0.05 (0.02)	0.0026	18	0.14%	6.54%	7
**Systolic blood pressure (per 10mmHg)**	−0.02 (0.01)	0.0026	19	0.14%	6.68%	8

Table shows the 19 predictors used as adjustment factors in the outcome analyses. *P*-value is from the *F* test in the final model. Factors available for inclusion in the model included main effects and first-order interactions for continuous, grouped, and data availability indicators as appropriate for the factors urine volume assayed, age (grouped factor has three levels only for interaction term), sex, treatment allocation, smoking (current smoker vs. not for interaction term), diabetes type and treatment, duration of diabetes, body mass index, systolic and diastolic blood pressures, reported treated hypertension, diabetic retinopathy, ethnicity, Townsend Index, aspirin use before screening, statin use at baseline, angiotensin-converting enzyme inhibitor use at baseline, baseline 5-year risk of serious vascular event, days sample spent in the post, total cholesterol, high-density lipoprotein cholesterol, non-high-density lipoprotein cholesterol, apolipoprotein A1, apolipoprotein B, glycated haemoglobin, urinary albumin-creatinine ratio, and estimated glomerular filtration rate.

^a^The factors listed as Step 0 were forced into the model.

**Figure 2:**
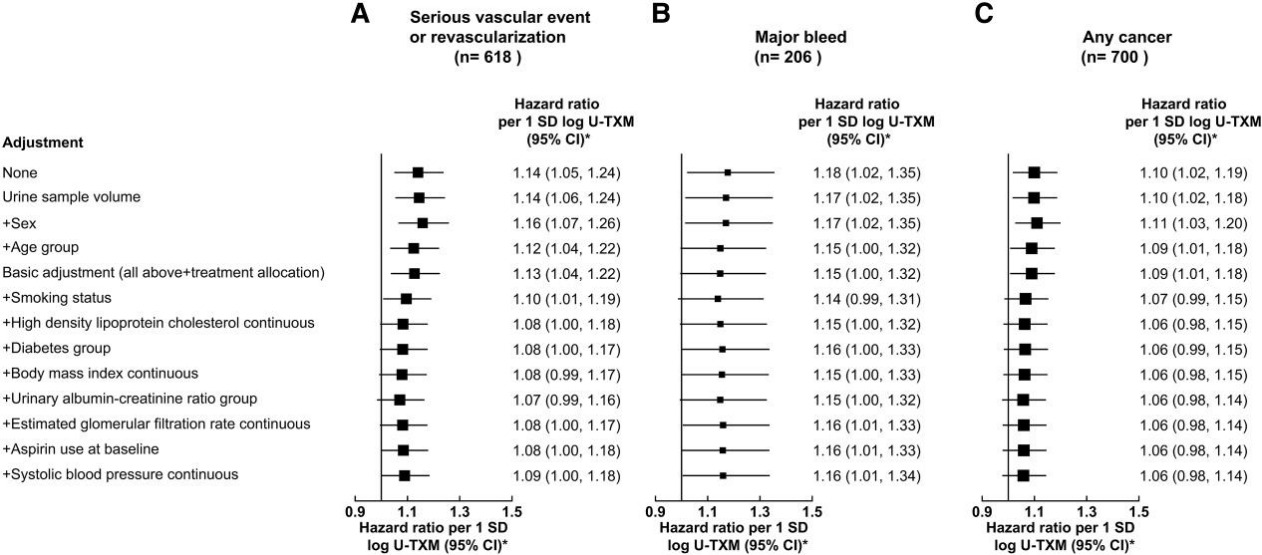
Linear associations of baseline (off-treatment) log urinary 11-dehydro-thromboxane B_2_ with the outcomes (Serious vascular event or revascularization [*A*], Major bleed [*B*] and Any cancer [*C*]) incrementally adjusting for predictors of log urinary 11-dehydro-thromboxane B_2_. A stepwise selection model was used to find baseline predictors of log urinary 11-dehydro-thromboxane B_2_ in both treatment arms. The basic adjustors age group, sex, urine volume assayed, and treatment allocation were forced into the model. The association of log urinary 11-dehydro-thromboxane B_2_ with outcome was then modelled by adjusting for the predictors of log urinary 11-dehydro-thromboxane B_2_, with the factors added incrementally in the order they were selected in the prediction model. *SD of log urinary 11-dehydro-thromboxane B_2_ is 0.622. Hence, 1 SD corresponds to an exp(0.622) = 1.862 fold higher urinary 11-dehydro-thromboxane B_2_. CI, confidence interval; *n*, number of events; SD, standard deviation; U-TXM, urinary 11-dehydro-thromboxane B_2_

**Figure 3:**
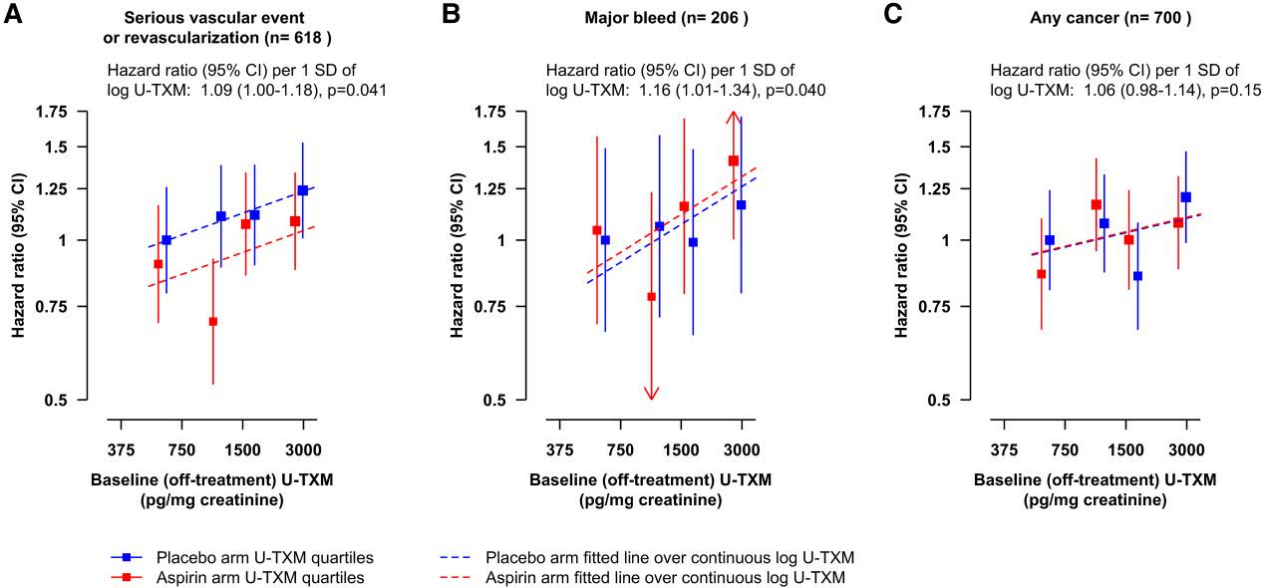
Associations of baseline (off-treatment) log urinary 11-dehydro-thromboxane B_2_ with outcomes (*A*, *B* and *C* as in Figure 2). Adjusted for treatment allocation, basic factors, and predictors of log urinary 11-dehydro-thromboxane B_2_. Model estimates and their floating absolute risk confidence intervals are plotted at the overall geometric mean urinary 11-dehydro-thromboxane B_2_ within quartile and randomized treatment allocation, with the placebo and aspirin arms offset by ±0.05 log urinary 11-dehydro-thromboxane B_2_ respectively to ensure the confidence intervals are distinguishable. The hazard ratio per 1 SD of log urinary 11-dehydro-thromboxane B_2_ is constrained to have the same slope in both placebo- and aspirin-allocated arms. SD of log urinary 11-dehydro-thromboxane B_2_ is 0.622. Hence, 1 SD corresponds to an exp(0.622) = 1.862 fold higher urinary 11-dehydro-thromboxane B_2_. CI, confidence interval; *n*, number of events; SD, standard deviation; U-TXM, urinary 11-dehydro-thromboxane B_2_

In analyses with basic adjustment, log U-TXM was marginally statistically significantly associated with SVE-R, with major bleeds, and with cancer (*Figure 2*). The HRs for SVE-R and cancer attenuated somewhat with increasing adjustment for predictors of U-TXM (particularly for current smoking status), whereas the HR for major bleed remained similar (*Figure 2*). After adjustment for the first 19 prediction parameters of log U-TXM, log U-TXM was still marginally statistically significantly associated with SVE-R (HR 1.09; 95% CI 1.00–1.18; *P* = .041) and major bleed (HR 1.16; 95% CI 1.01–1.34; *P* = .040) but was not statistically significantly associated with cancer (HR 1.06, 95% CI 0.98–1.14, *P* = .15) and showed no deviation from broad linearity for any outcome (*Figure 3*).

**Figure 4:**
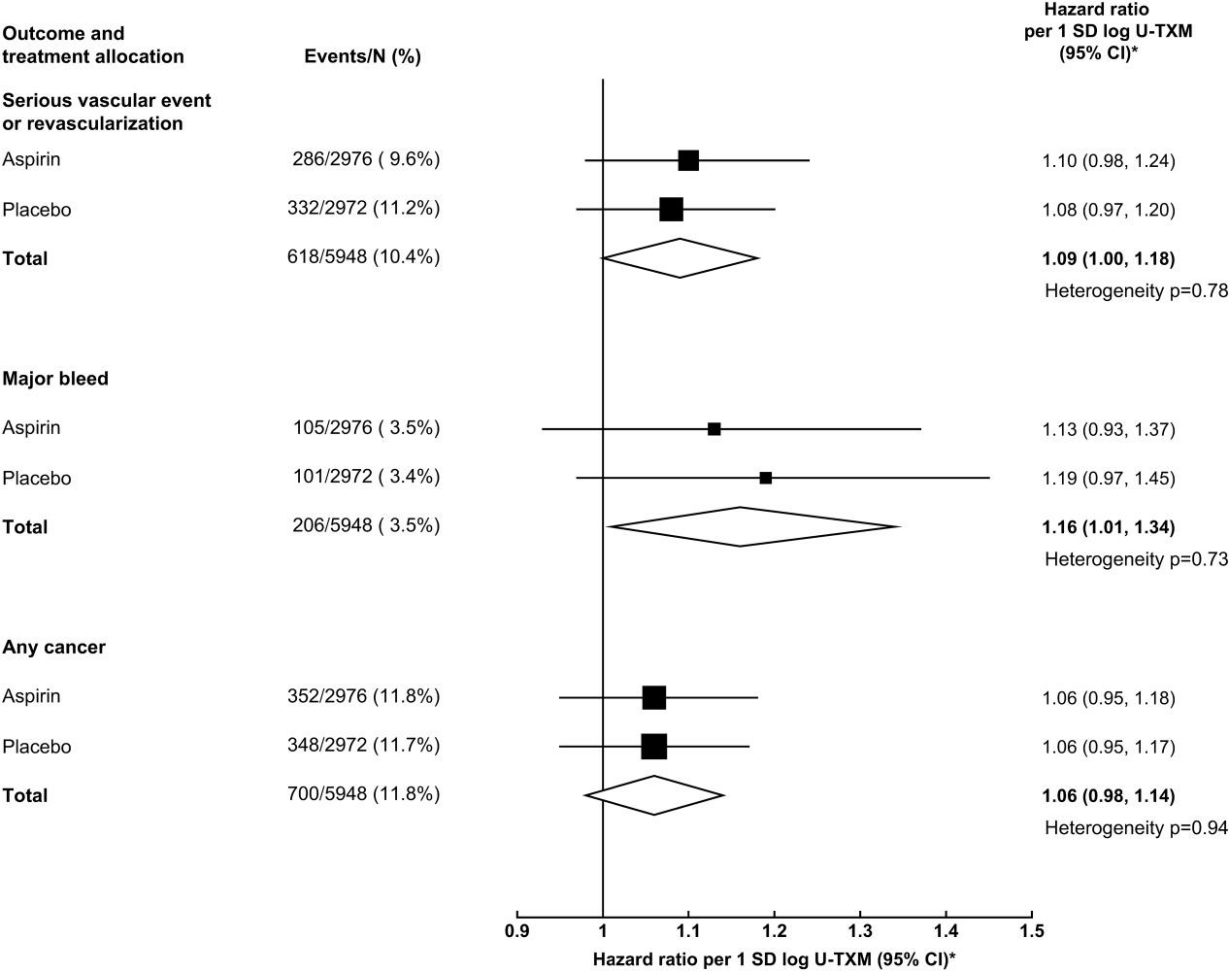
Linear associations of baseline (off-treatment) log urinary 11-dehydro-thromboxane B_2_ with outcomes in the aspirin- and placebo-allocated arms. Adjusted for basic factors and predictors of log urinary 11-dehydro-thromboxane B_2_. *SD of log urinary 11-dehydro-thromboxane B_2_ is 0.622. Hence, 1 SD corresponds to an exp(0.622) = 1.862 fold higher urinary 11-dehydro-thromboxane B_2_. CI, confidence interval; N, number of people; SD, standard deviation; U-TXM, urinary 11-dehydro-thromboxane B_2_

**Figure 5:**
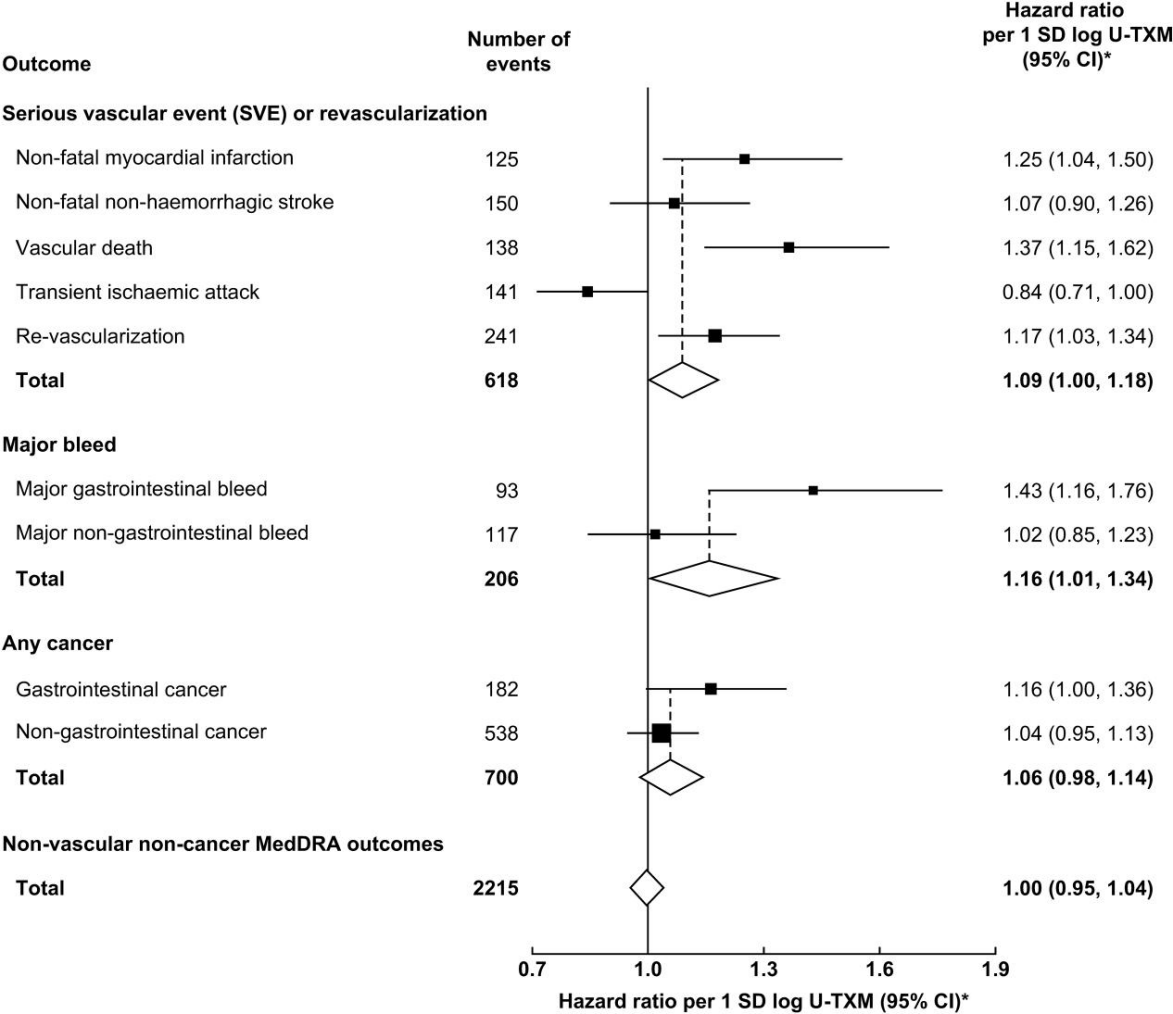
Linear associations of baseline (off-treatment) log urinary 11-dehydro-thromboxane B_2_ with constituents of the main outcomes and a composite of non-vascular, non-cancer outcomes classified by the Medical Dictionary for Regulatory Activities. Adjusted for treatment allocation, basic factors and predictors of log urinary 11-dehydro-thromboxane B_2_. *SD of log urinary 11-dehydro-thromboxane B_2_ is 0.622. Hence, 1 SD corresponds to an exp(0.622) = 1.862 fold higher urinary 11-dehydro-thromboxane B_2_. CI, confidence interval; MedDRA, Medical Dictionary for Regulatory Activities; SD, standard deviation; U-TXM, urinary 11-dehydro-thromboxane B_2_

There was no statistically significant heterogeneity in the strength of the associations by subsequent allocation to aspirin vs. placebo (*P* > .70; *Figure 4*). Furthermore, there was no heterogeneity in the effects of aspirin on outcomes by quartile of baseline U-TXM (*P* > .30; see Supplementary data online, *Figure S5*). Amongst the components of the main outcomes, the strongest association in the SVE-R outcome was observed for vascular death (HR 1.37; 95% CI 1.15–1.62), while the association between log U-TXM and major bleed appeared to be driven by gastrointestinal bleed (HR 1.43; 95% CI 1.16–1.76) (*Figure 5*). In a sensitivity analysis, no association was found between log U-TXM and a composite of non-vascular, non-cancer outcomes classified by MedDRA (HR 1.00; 95% CI 0.95–1.04; *Figure 5*).

**Figure 6:**
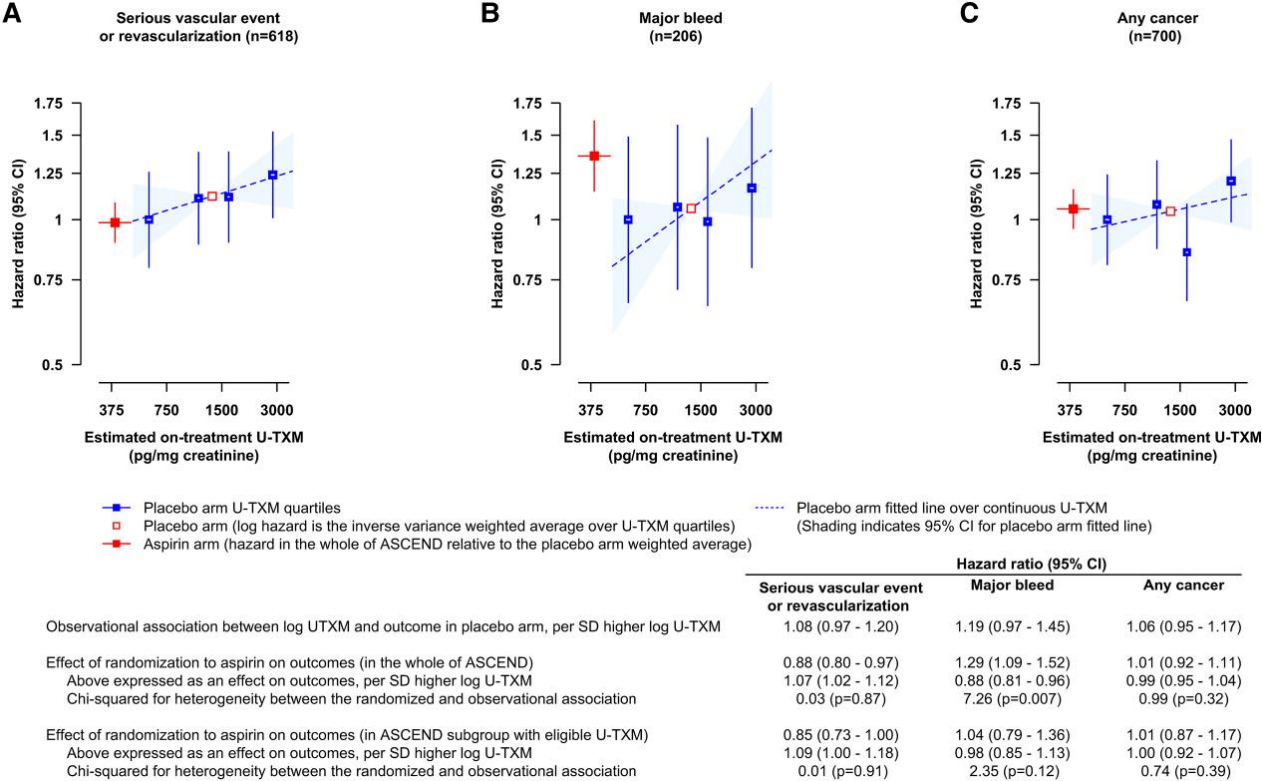
Comparison of the observational associations of log urinary 11-dehydro-thromboxane B_2_ with outcomes in the placebo arm and the implied effects in the randomized comparisons of aspirin vs. placebo, if the effect of aspirin on outcomes (*A*, *B* and *C* as in Figure 2) was mediated via its effect on TXA_2_ biosynthesis as reflected by log urinary 11-dehydro-thromboxane B_2_. Hazard ratios are plotted at the estimated on-treatment geometric mean urinary 11-dehydro-thromboxane B_2_, with the placebo groups plotted at the mean baseline log urinary 11-dehydro-thromboxane B_2_ within quartile (corresponding to geometric means of 601, 1120, 1634, and 2847 pg/mg creatinine). In a randomized comparison of urinary 11-dehydro-thromboxane B_2_ levels at a mean of 2 years after randomization in aspirin-allocated vs. placebo-allocated participants in ASCEND, aspirin was associated with 1.222 (1.023–1.421, *P* < .0001) lower log urinary 11-dehydro-thromboxane B_2_,^7^ and therefore, the hazard ratio in the aspirin arm relative to the placebo arm was plotted at the mean log urinary 11-dehydro-thromboxane B_2_ in the placebo arm, 7.193, minus 1.222 = 5.971 (corresponding to 392 pg/mg creatinine). The 95% confidence intervals for the mean urinary 11-dehydro-thromboxane B_2_ are visible for the aspirin arm but small and within the boxes for the placebo arm. The observational analysis in the placebo arm was from a model including both arms, adjusted for treatment allocation, basic factors and predictors of log urinary 11-dehydro-thromboxane B_2_ and with different slopes allowed for the hazard ratio per 1 SD of log urinary 11-dehydro-thromboxane B_2_ in the placebo and aspirin allocated arms. SD of overall baseline log urinary 11-dehydro-thromboxane B_2_ is 0.622. Hence, 1 SD higher log urinary 11-dehydro-thromboxane B_2_ corresponds to an exp(0.622) = 1.862 fold higher U-TXM. CI, confidence interval; *n*, number of events; SD, standard deviation; U-TXM, urinary 11-dehydro-thromboxane B_2_

The secondary analysis shown in *Figure 6* compares the observational associations between log U-TXM and outcomes in the placebo arm with the implied effects in the randomized comparisons if the effect of aspirin treatment on outcomes was mediated via its effect on platelet TXA_2_ biosynthesis, as reflected by log U-TXM. Randomization to aspirin was assumed to lower log U-TXM by 1.96 SD.^7^ The statistically significant 12% (95% CI 3%–20%) proportional reduction in SVE-R with aspirin in all 15 480 participants randomized in ASCEND^12^ corresponds to 7% (95% CI 2%–12%) increase per SD higher log U-TXM, which is very similar to the observational association.

In the randomized comparison of allocation to aspirin vs. placebo in all 15 480 ASCEND participants, there was a 29% (95% CI 9%–52%) proportional increase in the risk of major bleeds based on 559 events in the whole of ASCEND,^12^ which corresponds to a 12% (4%–19%) reduction per SD higher log U-TXM. The observational effect of log U-TXM was weakly in the opposite direction and not consistent with this randomized effect. However, there were only 206 major bleeds in the present observational study of U-TXM excretion and so the CIs were wide, and furthermore, in this subgroup of patients, there was only a 4% increase in major bleeds with allocation to aspirin (95% CI −21% to 36%). Thus, the data on bleed are too limited for unequivocal interpretation.

Neither the randomized comparison of aspirin vs. placebo nor the observational analysis of U-TXM showed any statistically significant association with cancer.

## Discussion

In a cohort of almost 6000 people with mostly type 2 diabetes mellitus without diagnosed cardiovascular disease, baseline rates of U-TXM excretion, largely reflecting platelet TXA_2_ biosynthesis, were marginally significantly associated with a detectable increase in risk of SVE-R (HR per 1 SD higher log U-TXM 1.09; 95% CI 1.00–1.18). This was very similar to the implied effect of subsequent randomization to aspirin if its statistically significant effect on SVE-R was mediated via its effect on platelet TXA_2_ biosynthesis, as reflected by U-TXM. By contrast, the weakly positive observational association of U-TXM with major bleed was apparently not consistent with the protective effect of higher U-TXM implied by the randomized comparisons (*Figure 6*). No significant associations were seen between the measured U-TXM excretion rate and subsequent risk of cancer (*Figure 6 and Structured Graphical Abstract*).

The relatively small number of bleeds in those with a U-TXM measurement (approximately 1/3 of SVE-R) and the lack of a significant excess of bleeds with aspirin allocation in this sub-cohort of patients make any inference from the observations on major bleed questionable. However, a haemostatic imbalance triggered by high platelet activation cannot be excluded. In particular, the highest platelet activation rate *in vivo* can trigger consumption of primary haemostatic factors (e.g. von Willebrand factor) that in turn can contribute to a paradoxical increase in mucosal bleeding in addition to thrombosis, as occurs in thrombocytotic myeloproliferative neoplasms, and Heyde's syndrome.^27–29^ Thus, it is biologically plausible that the relationship between the degree of platelet activation and bleeding could be non-linear, with bleeding associated with the extremes of U-TXM values due to severely reduced or maximally enhanced platelet function *in vivo*. Previously, Poisson regression in 93 918 individuals without known vascular disease at entry within aspirin primary prevention trials indicated that the main risk factors for coronary events were also associated with haemorrhagic events.^30^

A recent prospective observational study of 3044 participants [amongst whom 1680 (55%) were not on aspirin at study entry] in the Framingham Heart Study followed over a median of 11.9 years showed that in those not using aspirin, when adjusted for known cardiovascular risk factors, U-TXM was positively associated with cardiovascular death (HR 2.82; 95% CI 1.39–5.66; *P* < .004 between quartiles 4 and 1–3), but this was based on just 45 cardiovascular deaths with no information on non-fatal events.^21^ No association was seen in aspirin users between U-TXM and cardiovascular death unlike in the two studies described below, although there was a significant association with total mortality. In contrast to the ASCEND data, this study also reported a significant association between U-TXM and cancer death in those not on aspirin, but the degree to which this association is adjusted for potential confounders is not clear.

Two earlier sub-studies of randomized trials assessed the association of U-TXM excretion at study entry and risk of future cardiovascular events amongst people taking aspirin and found stronger associations between U-TXM and vascular events in comparison with the present study.^31,32^ In the Heart Outcomes Prevention Evaluation (HOPE) study, U-TXM at baseline was measured in 488 aspirin-treated cases who had a composite of either myocardial infarction, stroke, or cardiovascular death during 5 years of follow-up and 488 aspirin-treated sex- and age-matched subjects without an event.^31^ After adjustment for baseline differences, the odds for the composite outcome increased with each increasing quartile of U-TXM, with patients in the highest quartile (U-TXM > 33.8 ng/mmol creatinine) having a 1.8-times-higher odds than those in the lowest quartile (U-TXM < 15.1 ng/mmol creatinine).^31^ The association with bleeding outcomes was not reported. Similarly, in the Clopidogrel for High Atherothrombotic Risk and Ischemic Stabilization, Management and Avoidance (CHARISMA) trial, U-TXM excretion was measured in 3261 aspirin-treated patients and U-TXM in the highest quartile was associated with a higher risk of stroke, myocardial infarction, or cardiovascular death compared with the lowest quartile (HR 1.66). They also reported a non-significant trend towards a higher risk of bleeding with higher U-TXM.^32^ However, in both these studies, higher rates of U-TXM excretion may have reflected inadequate adherence to aspirin therapy at the time of sampling and/or impaired antiplatelet pharmacodynamics, or unreported NSAID intake, precluding unequivocal interpretation of these findings. The standard deviation of log U-TXM was ∼0.7 in CHARISMA, similar to that in ASCEND. Therefore, these findings seem to correspond to a considerably stronger association between the TXA_2_ generated and cardiovascular events than that observed in ASCEND despite the use of once-daily low-dose aspirin. The consistency amongst these large prospective data may imply incomplete suppression of platelet COX-1 by a standard once-daily dose in at least a proportion of the patients.^33,34^ An alternative, but less likely, explanation of these findings is that there is an important contribution from extra-platelet sources of TXA_2_. Moreover, a recent study has suggested that aspirin in primary prevention may have a greater effect in individuals homozygous for the *GUCY1A3* risk (G) allele, which has been shown to modify the inhibition of nitric oxide on platelets and increase cardiovascular disease.^35^ Genotyping was not available in ASCEND but establishing whether these homozygote patients also have higher U-TXM levels while on aspirin could help clarify the pathways of action.

The strengths of the ASCEND sub-study are as follows: (i) it included a prospective measurement of U-TXM excretion rate in people not taking either aspirin or a NSAID; (ii) it included almost 6000 participants amongst whom >600 experienced an SVE-R during more than 6 years of follow-up, a larger number than in either of the two earlier studies (488 in HOPE and 144 in CHARISMA); (iii) it included a relatively homogeneous population of subjects with diabetes mellitus, a clinical setting in which enhanced TXA_2_ biosynthesis has been previously characterized,^13,18^ in contrast to the heterogeneous mix of high-risk patients enrolled in the other sub-studies; (iv) information was available on a large number of potential confounding factors allowing for detailed adjustment; and (v) it was able to undertake a sensitivity analysis looking at the association between U-TXM and all non-cardiovascular, non-bleeding, and non-cancer reported serious adverse events demonstrating no association, implying that the observed effects cannot readily be accounted for by confounding with general poor health.

Possible limitations include a single measure of U-TXM excretion that may not accurately reflect the extent of platelet activation during long-term follow-up. However, in a 3-year study of 118 subjects with type 2 diabetes mellitus, repeated measurements of U-TXM excretion appeared relatively stable over time within subjects, independent of disease duration.^36^ The variability in both the U-TXM and creatinine measures (U-TXM final values are corrected for concentration of urinary creatinine) will have reduced the power to detect effects and will have led to an underestimation of the strength of any true association with SVE-Rs. Also, the potential contribution of extra-platelet sources of TXA_2_ biosynthesis to U-TXM excretion cannot be excluded, as urinary prostanoid metabolites do not reflect a single specific site of origin of the parent compound.^2^ However, other data are consistent with platelets providing the main source of TXA_2_ biosynthesis in this setting.^13^ In addition, the association of U-TXM measures with a range of cardiovascular risk factors supports the hypothesis that enhanced platelet TXA_2_ biosynthesis may represent a common pathophysiologic mechanism underlying the vascular impact of various risk factors on cardiovascular events.^8^

Despite these shortcomings, the effect size of increasing TXA_2_ biosynthesis on the risk of SVE-R appears biologically plausible. If the observed associations are causal, they are consistent with the moderate treatment effect of low-dose aspirin in the whole ASCEND trial.^11,12^ The practical clinical readout is the possibility of using U-TXM as a biomarker to individualize and refine aspirin dosing in order to achieve an optimal inhibition of platelet activation. Data also suggest that an adequate control of U-TXM by aspirin may paradoxically also control the gastrointestinal bleeding risk. Prior evidence from the effects of aspirin on log U-TXM and on SVE-R reduction in ASCEND^7^ suggests about a 7% proportional increase in events per 1 SD higher log U-TXM (*Figure 6*). The observational association between log U-TXM and SVE-R in this sub-study was consistent with this finding. Further data are needed to confirm whether the U-TXM levels are a useful predictor of cardiovascular risk in other clinical settings and whether genetic factors have any influence on the efficacy of aspirin in diabetes.

Observational studies and long-term follow-up of some randomized trials, with lengths of follow-up ranging from 11 to over 20 years, suggest that aspirin treatment may protect against particular cancers, the strongest evidence being for gastrointestinal cancers.^37–42^ However, recent long-term large randomized trials have shown no emerging effect on cancer risk.^12,43,44^ Several lines of evidence are consistent with the hypothesis that activated platelets at sites of mucosal injury contribute to colorectal tumourigenesis and metastatization through direct cell–cell interactions and the release of different mediators, including TXA_2_.^9^ However, with the limited 7.4 years of follow-up available to date, neither this ASCEND sub-study nor the randomized data on cancer incidence provide support that aspirin is protective against cancer by its anti-platelet effect, but any protective effects of aspirin may take many years to become apparent.^45^ Pre-specified analyses of cancer in ASCEND are planned for 5 and 10 years after the end of the scheduled treatment period to re-assess this based on longer-term follow-up.

In conclusion, this large, prospective study shows that amongst adults with diabetes but no evident cardiovascular disease, U-TXM excretion, a non-invasive biomarker of TXA_2_-mediated platelet activation *in vivo*, was marginally associated with subsequent risk of a composite outcome of SVE-R, independent of other cardiovascular risk factors. This association with SVE-R is consistent with the involvement of platelet TXA_2_ production in diabetic atherothrombosis, but more data are needed to confirm this and to clarify its potential role in bleeding risk.

## Supplementary Material

ehad868_Supplementary_Data

## Declarations

### Data Availability

Requests for data sharing will be handled in line with the data access and sharing policy of the Nuffield Department of Population Health, University of Oxford (https://www.ndph.ox.ac.uk/files/about/ndph-data-access-policy-1.pdf).

### Pre-registered Clinical Trial Number

The pre-registered clinical trial number is ISRCTN.com: ISRCTN60635500; ClinicalTrials.gov: NCT00135226OR.
