# Supplementary material for: Thromboxane biosynthesis and future events in diabetes: the ASCEND trial
Source: Eur Heart J. 2024 Feb 22;45(15):1355–67. doi: 10.1093/eurheartj/ehad868 (PMC11015956; doi:10.1093/eurheartj/ehad868)
Supplement: ehad868_Supplementary_Data [file ehad868_supplementary_data.pdf]

Supplemental materials

Table of contents

Supplemental Materials and Methods 2

Supplemental Results 3

Supplemental Figure Legends 4

Supplemental References 5

Supplemental Tables

Table S1 Baseline characteristics by availability of a valid urinary 11-dehydro thromboxane B2 (U-TXM) measure in the present and previous ASCEND sub-studies 7

Supplemental Figures

Figure S1 Recovery of spiked 11-dehydro thromboxane B2 (TXM) standard from urine samples 8

Figure S2 Reproducibility of ELISA measurements 9

Figure S3 Reproducibility of the extraction method 10

Figure S4 Reproducibility of urinary creatinine measurements 11

Figure S5 Associations of aspirin versus placebo treatment allocation with outcomes in U-TXM quartiles. 12

Supplemental Materials and Methods

Materials used for 11-dehydro thromboxane B_2 (TXM) extraction and assay

The following materials were used (in alphabetical order): Acetic acid CARLO ERBA Reagents S.r.l., Milan, Italy; Acetonitrile, Panreac Química SLU; Column Octadecyl (C18) 1 ml / 50 mg, Bakerbond-spe, JT Baker Inc, Phillipsburg, NJ, USA, Column Silica Gel 1 ml / 100 mg, Bakerbond-spe, JT Baker Inc, Creatinine Colorimetric Detection Kit, Enzo Life Sciences, Farmingdale, NY, USA; Creatinine Standard, Cayman Chemicals, Ann Arbor, MI, USA; 11-dehydro-TXB_2 EIA Antiserum, Cayman Chemicals; TXM EIA antiserum L16-R5, non-commercial(1), TXM AChE Tracer, Cayman Chemicals; TXM EIA Standard, Cayman Chemicals; TXM, Cayman Chemicals; ELISA plates Uncoated Nunc MaxiSorp, Thermo Fischer Scientific Nunc A/S, Roskilide, Denmark; Ethylacetate, CARLO ERBA Reagents S.r.l.; ^{3}H-thromboxane B_2 7.7, TBq / mmol, GE Healthcare Amersham, Buckinghamshire, UK; Instagel-PLUS, Perkin Elmer Italia, Milan, Italy; Isooctane CARLO ERBA Reagents S.r.l; Methanol, CARLO ERBA Reagents S.r.l.; Mouse monoclonal anti Rabbit IgG, Cayman Chemicals.

Urine extraction

Urine samples of 1 mL were thawed at 37°C and centrifuged at 340*g* for 10 minutes; 60 ul of acetic acid was added to reach a pH of approximately 3.5, 1,000 cpm ^{3}H-thromboxane B_2 were added to 1 mL urine sample. Samples were loaded into a 1 mL/50 mg C18 column, prewashed with 1 mL absolute methanol and 2.5 mL distilled H_2O. Column was then washed with 1.6 mL distilled H_2O and 2 mL acetonitrile/water (15:85, vol/vol), dried and eluted with 2.5 mL isooctane/ethyl acetate (1:1, vol/vol). The eluate was loaded on a 1 mL/100 mg SiOH column prewashed with 2 mL isooctane/ethyl acetate (1:1,vol/vol), eluted with 2 mL ethylacetate/methanol (60:40, vol/vol). After drying, eluate was resuspended in 1 mL PBS/0.1% BSA buffer, 500ul of the final resuspension were counted for calculating the recovery. The ELISA measurements were corrected for the % of recovery based on ^{3}H-thromboxane B_2 cpm counts. This method has been published before (2). For urine samples of <1ml volume, 0.5 mL were extracted, by adapting the above protocol as follows: 30 ul acetic acid were added to 0.5 mL urine, after drying, the eluate was resuspended in 0.5 mL PBS/0.1% BSA buffer, 250ul were counted for assessing recovery.

To assess the stability and reproducibility of the extraction procedure over the entire sub-study duration, a pool of urine from healthy donors was aliquoted into 1mL samples and frozen until use. In each experimental set, aliquots were thawed and spiked with vehicle (ethanol 0.05% vol/vol) or with known concentrations of the exogenous cold TXM commercial standard at final concentrations ranging from 0.5 and 2 ng/ml. These experiments were performed at least every two months over the entire duration of the analyses.

TXM measurements

Urinary TXM was measured in the extracted samples by a standard Enzyme Linked Immunosorbent Assay (ELISA) assay (2,3). Ninety-six-well plates were coated with commercial monoclonal anti-rabbit IgG antibodies according to the standard method for coating. TXM in urinary extracts was measured with a standard AchE ELISA immunometric method, using a specific rabbit polyclonal antibody (1). The ELISA assay using this antibody had a range of detection from 0.5 to 0.0039 ng/ml, a sensitivity calculated as B/B_0 (Bound/Maximum Bound) 80% of 0.01 ng/ml, and an overall inter-assay coefficient of variation of 8.8%. Samples that measured <0.018 ng/ml were assayed with a different commercial anti-rabbit IgG, which had a lower range of detection from 0.25 to 0.0019 ng/ml and a 80% B/B0 of 0.004 ng/ml. The cross-reactivity of the anti-TXM antibodies against other prostanoids that can be measured in urines, namely PGE_2 and the isoprostane 8-iso-PGF_{2alpha} was <0.05%.

The validation of the method was based on the U.S. Food and Drug Administration guidelines for Validation of Bioanalytical Methods (4). Internal standards were used for intra-assay validation and consisted of pools of urinary extracts from healthy donors which were aliquoted, frozen and one aliquot was used per each plate.

TXM final values were corrected for the concentration of urinary creatinine, that was measured by a commercial kit based on the Jaffe's reaction (5). Two internal standards were used in each assay plate: a pool of urine and a commercial standard. These internal standards were always included to assess the consistency and reproducibility of the assays over time.

Supplemental Results

Reproducibility of Urinary Extraction method

To assess the stability and reproducibility of the extraction procedure, we performed experiments with cold TXM spike during the entire study duration. Representative experiments

are shown on Figure S1. Cold spike recoveries appeared quite consistent over time and independent of the endogenous levels of TXM in the spiked urine samples.

Reproducibility of ELISA measurements in urinary extracts

As part of the validation process, we repeated the ELISA assay in 320 urine extracts selected based on residual sample volume availability. The correlation between the two measurements is shown in Figure S2.

Reproducibility of the urine extraction procedure

We also assessed the reproducibility of the urine extraction procedure and ELISA measurements in 319 urinary samples selected based on the remaining available volume. The second extraction was always performed with 5 ml urine. A high correlation about the two measurements is represented in Figure S3.

Reproducibility of urinary creatinine measurements

Urinary creatinine was repeated in 163 samples, randomly selected based on the available remaining volume. The correlation between the two measurements was very high and shown in Figure S4.

Supplemental Figure Legends

Figure S1. Recovery of spiked 11-dehydro thromboxane B_2 (TXM) standard from urine samples. Urine samples from healthy volunteers were spiked with exogenous TXM or vehicle, extracted by chromatography and analysed. The y-intercept corresponds to the amount of TXM in the vehicle-spiked urine sample. Each line represents the fitting of a representative experimental set at different study months. The two panels show different study months.

Figure S2. Reproducibility of ELISA measurements. In 320 random samples, urinary extracts were measured by the ELISA on two distinct occasions. The plot represents the values of 11-dehydro thromboxane B_2 (TXM) of the 1st and the 2nd determination, the linear regression line and 95% confidence interval. TXM is expressed in pg/ml of urine.

Figure S3. Reproducibility of the extraction method. Extractions were repeated from 319 urine samples, and 11-dehydro thromboxane B_2 (TXM) was measured in the repeated extracts. The plot represents the values of urinary TXM corrected by % recovery in the 1st and in the 2nd extraction, the linear regression line and 95% confidence interval. TXM is expressed in pg/ml of urine.

Figure S4. Reproducibility of urinary creatinine measurements. In 163 random samples, urinary creatinine analyses were assessed on two separate occasions. The plot represents the 1st and the 2nd determinations, the linear regression line and 95% confidence interval.

Figure S5. Associations of aspirin versus placebo treatment allocation with outcomes in U-TXM quartiles. Adjusted for basic factors and predictors of log U-TXM. P-value for trend over quartiles is calculated using the mean log U-TXM within each quartile. CI=confidence interval, N=number of participants.

Supplemental References

1. Pradelles P, Grassi J, Maclouf J. Enzyme immunoassays of eicosanoids using acetylcholine esterase as label: an alternative to radioimmunoassay. *Anal Chem.* 1985;57:1170-3.
2. Pagliaccia F, Habib A, Pitocco D et al. Stability of urinary thromboxane A2 metabolites and adaptation of the extraction method to small urine volume. *Clin Lab.* 2014;60:105-11.
3. Wang Z, Ciabattoni G, Creminon C et al. Immunological characterization of urinary 8-epi-prostaglandin F2 alpha excretion in man. *J Pharmacol Exp Ther.* 1995;275:94-100.
4. U.S. Department of Heath and Human Services FaDA, Center for Drug Evaluation and Research. Guidance for Industry: Bioanalytical Method Validation. 2001. Accessed 30 Jan 2023.

https://www.moh.gov.bw/Publications/drug_regulation/Bioanalytical%20Method%20Validation%20FDA%202001.pdf.

5. Cook JG, Association of clinical Biochemists S, Technica C. Factors influencing the assay of creatinine. *Ann Clin Biochem.* 1975;12:219-32.

Table S1: Baseline characteristics by availability of a valid urinary 11-dehydro thromboxane B2 (U-TXM) measure in the present and previous ASCEND sub-studies

	U-TXM study	
**Characteristic**	**Present study N (%)**	**Previous study* N (%)**
Overall	5948	152
Age at randomization (years)		
<60	1558 (26.2%)	36 (23.7%)
≥60, <70	2615 (44.0%)	68 (44.7%)
≥70	1775 (29.8%)	48 (31.6%)
Sex		
Male	3802 (63.9%)	103 (67.8%)
Female	2146 (36.1%)	49 (32.2%)
Randomized allocation		
Placebo	2972 (50.0%)	76 (50.0%)
Aspirin	2976 (50.0%)	76 (50.0%)
Body mass index (kg/m^2)		
<25	863 (14.5%)	28 (18.4%)
≥25, <30	2177 (36.6%)	57 (37.5%)
≥30	2709 (45.5%)	63 (41.4%)
Unknown	199 (3.3%)	4 (2.6%)
Type of diabetes		
Type 1	214 (3.6%)	11 (7.2%)
Type 2	5734 (96.4%)	141 (92.8%)
Diabetes management in type 2 diabetes		
Diet	1021 (17.8%)	26 (18.4%)
Oral hypoglycemic	3673 (64.1%)	82 (58.2%)
Insulin	1040 (18.1%)	33 (23.4%)
Smoking status		
Current	396 (6.7%)	9 (5.9%)
Former	2854 (48.0%)	74 (48.7%)
Never	2619 (44.0%)	68 (44.7%)
Unknown	79 (1.3%)	1 (0.7%)
Baseline 5-year risk of serious vascular event		
Low (<5%)	2122 (35.7%)	49 (32.2%)
Moderate (≥5%, <10%)	2642 (44.4%)	72 (47.4%)
High (≥10%)	1184 (19.9%)	31 (20.4%)
Nonsteroidal anti-inflammatory (NSAID) drugs at baseline		
No	5948 (100.0%)	138 (90.8%)
Yes	0 (0.0%)	14 (9.2%)
High density lipoprotein cholesterol (mmol/L)		
< 1.0	1228 (20.6%)	31 (20.4%)
≥1.0, < 1.5	3439 (57.8%)	92 (60.5%)
≥1.5	1233 (20.7%)	28 (18.4%)
No Result Available	48 (0.8%)	1 (0.7%)
Estimated glomerular filtration rate (ml/min/1.73m^2)		
< 60	762 (12.8%)	18 (11.8%)
≥ 60, < 90	2382 (40.0%)	70 (46.1%)
≥ 90	2756 (46.3%)	63 (41.4%)
No Result Available	48 (0.8%)	1 (0.7%)
Urinary albumin-creatinine ratio (mg/mmol)		
< 3	5135 (86.3%)	138 (90.8%)
≥3	808 (13.6%)	14 (9.2%)
No Result Available	5 (0.1%)	0 (0.0%)

N=Number, CI=Confidence interval.
*Parish S et al. 'Effect of low-dose aspirin on urinary 11-dehydro-thromboxane B2 in the ASCEND (A Study of Cardiovascular Events iN Diabetes) randomized controlled trial'. Trials (2023)

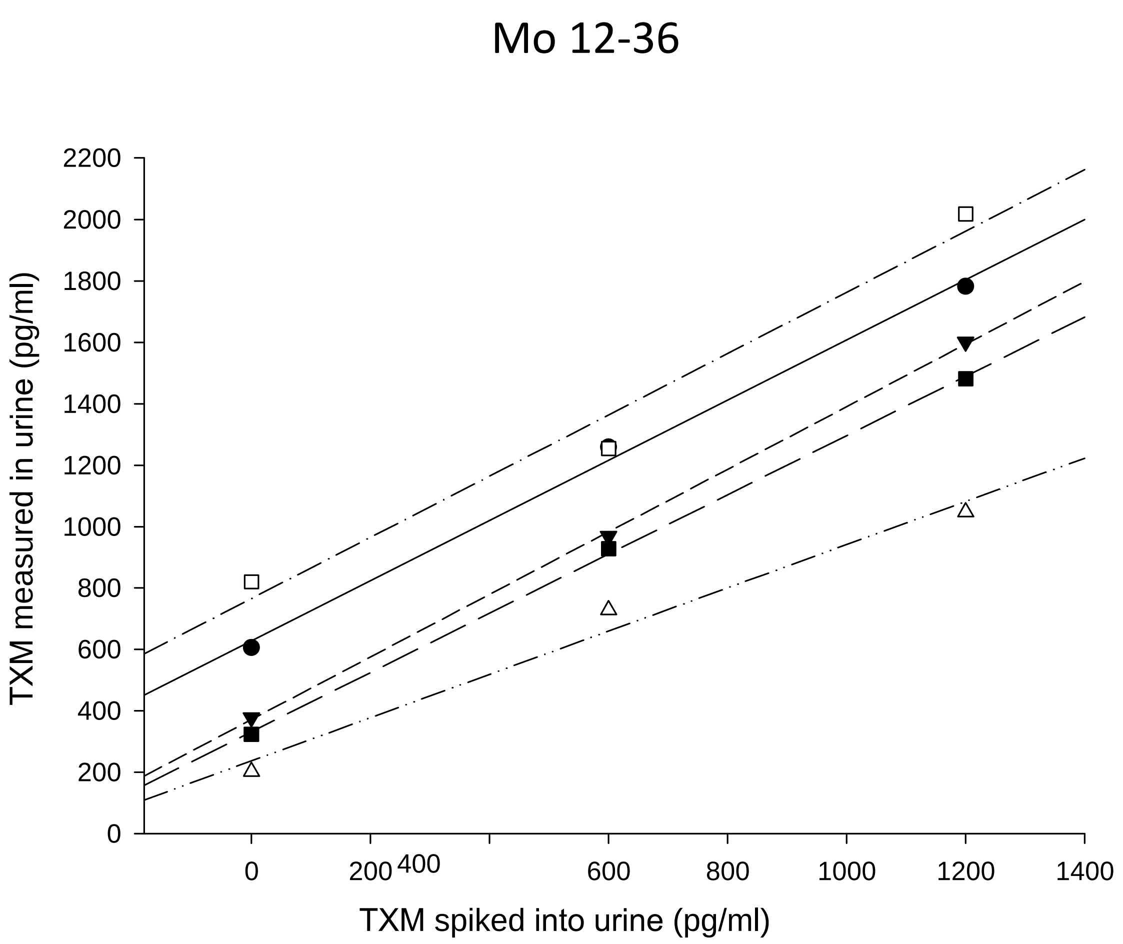
Mo 12-36
TXM measured in urine (pg/ml)
0
200
400
600
800
1000
1200
1400
1600
1800
2000
2200
0
200
400
600
800
1000
1200
1400
TXM spiked into urine (pg/ml)

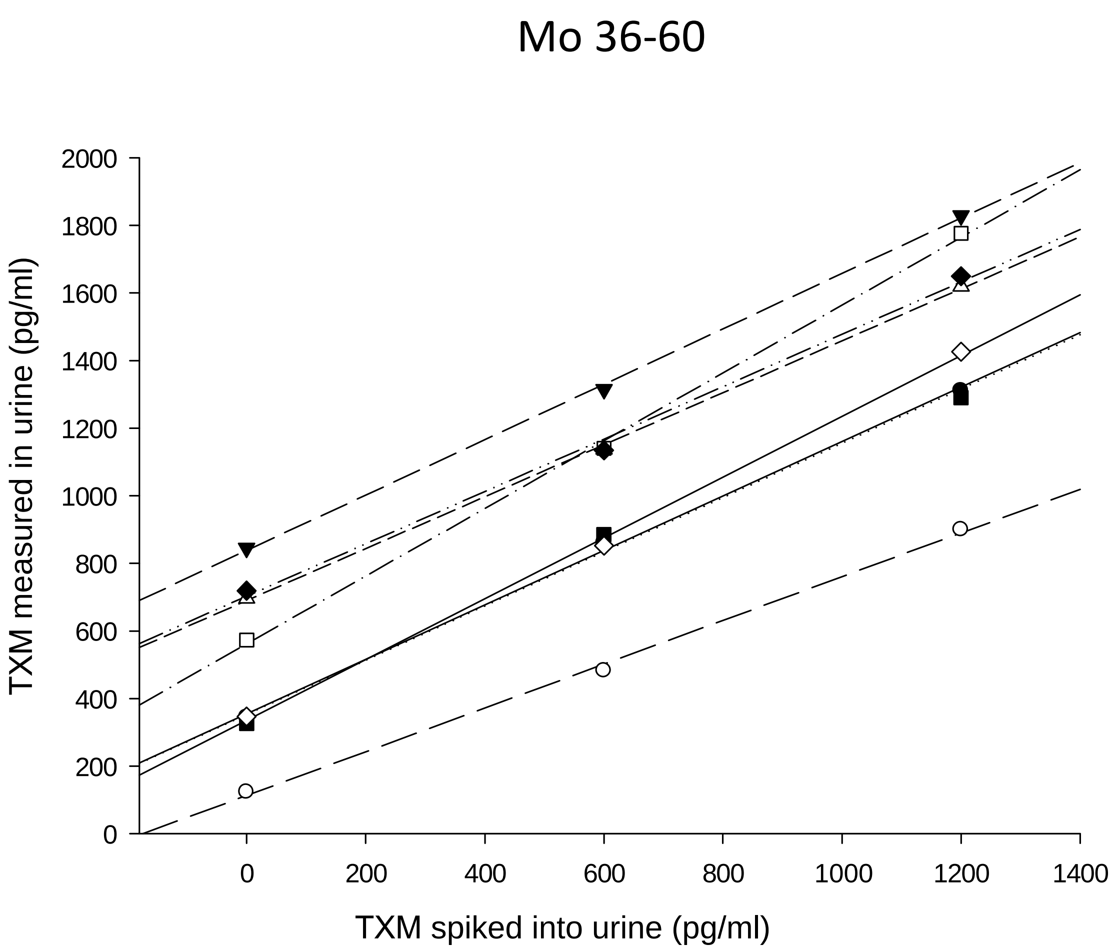
Mo 36-60
TXM measured in urine (pg/ml)
0
200
400
600
800
1000
1200
1400
1600
1800
2000
0
200
400
600
800
1000
1200
1400
TXM spiked into urine (pg/ml)

Figure S1

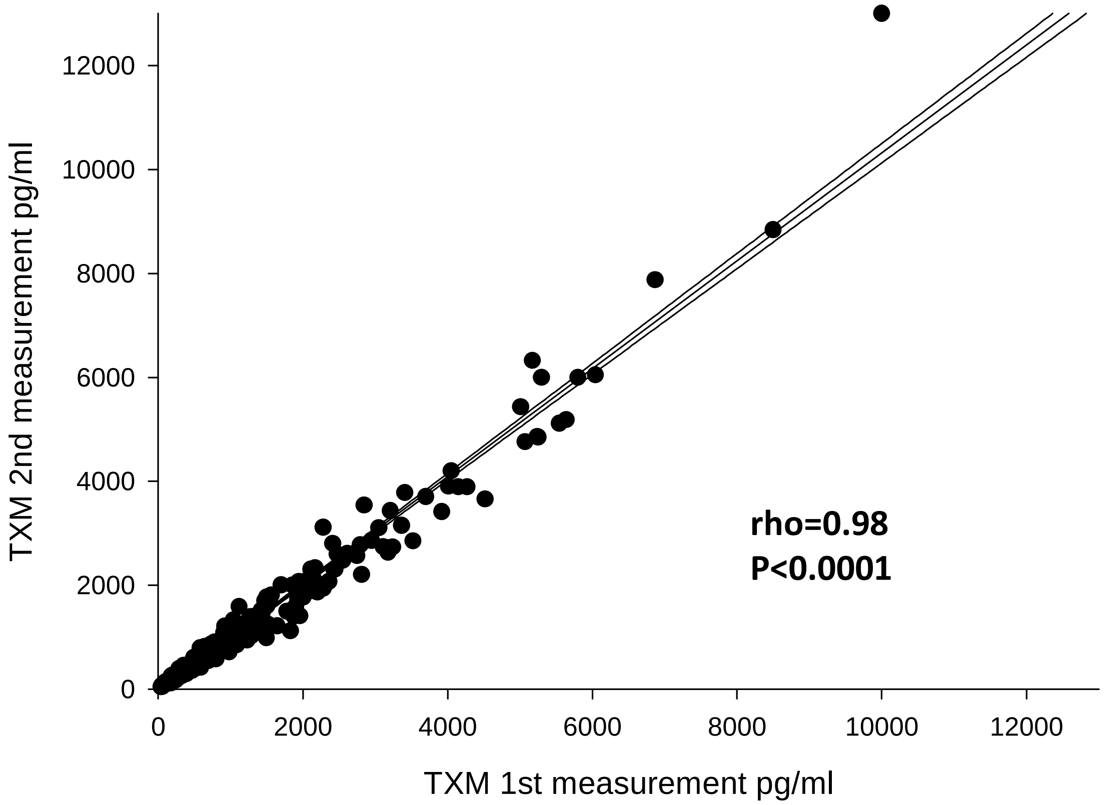
12000
10000
8000
6000
4000
2000
0
TXM 2nd measurement pg/ml
0
2000
4000
6000
8000
10000
12000
TXM 1st measurement pg/ml
rho=0.98
P<0.0001

Figure S2

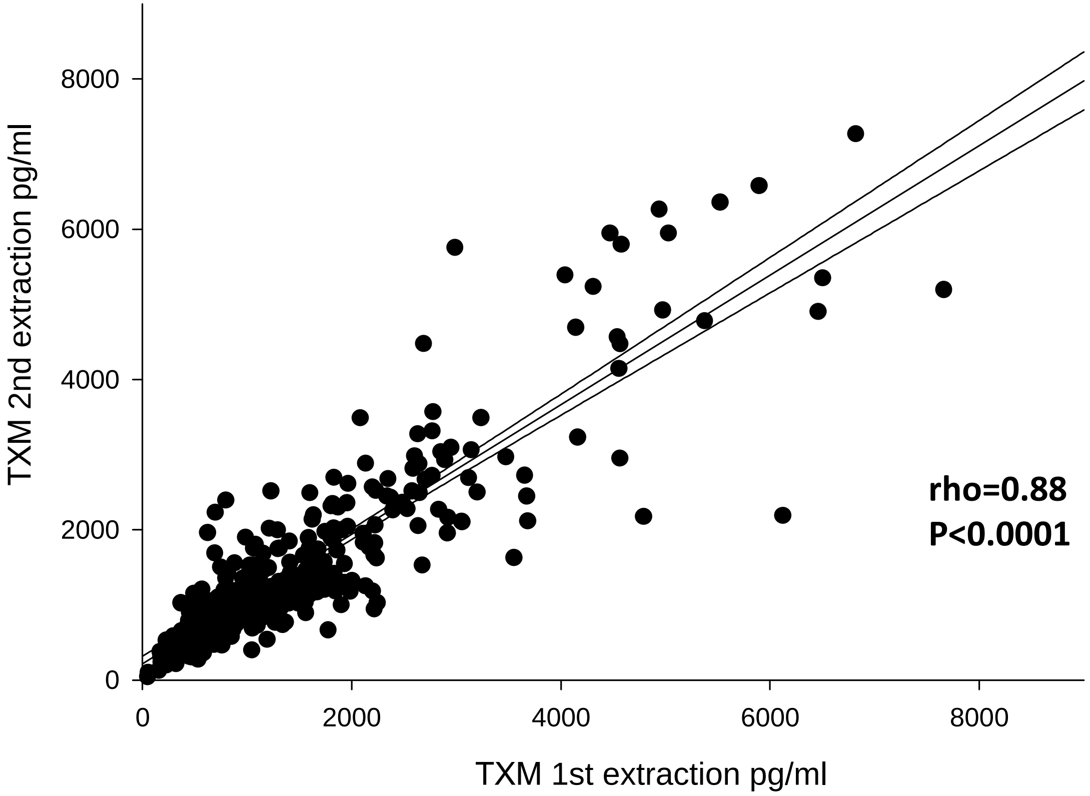
TXM 2nd extraction pg/ml
8000
6000
4000
2000
0
0
2000
4000
6000
8000
TXM 1st extraction pg/ml
rho=0.88
P<0.0001

Figure S3

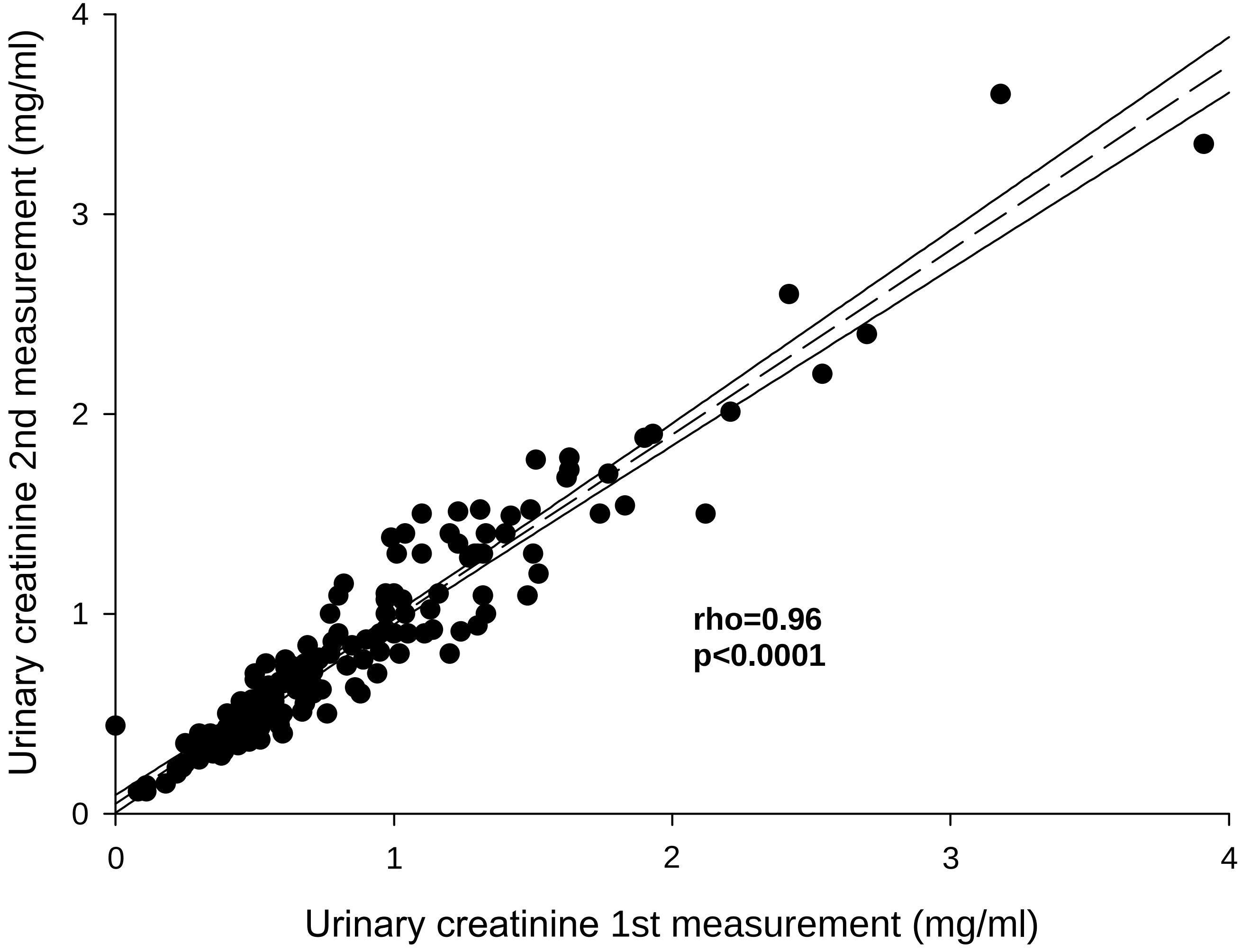
Urinary creatinine 2nd measurement (mg/ml)
Urinary creatinine 1st measurement (mg/ml)
0
1
2
3
4
rho=0.96
p<0.0001

Figure S4

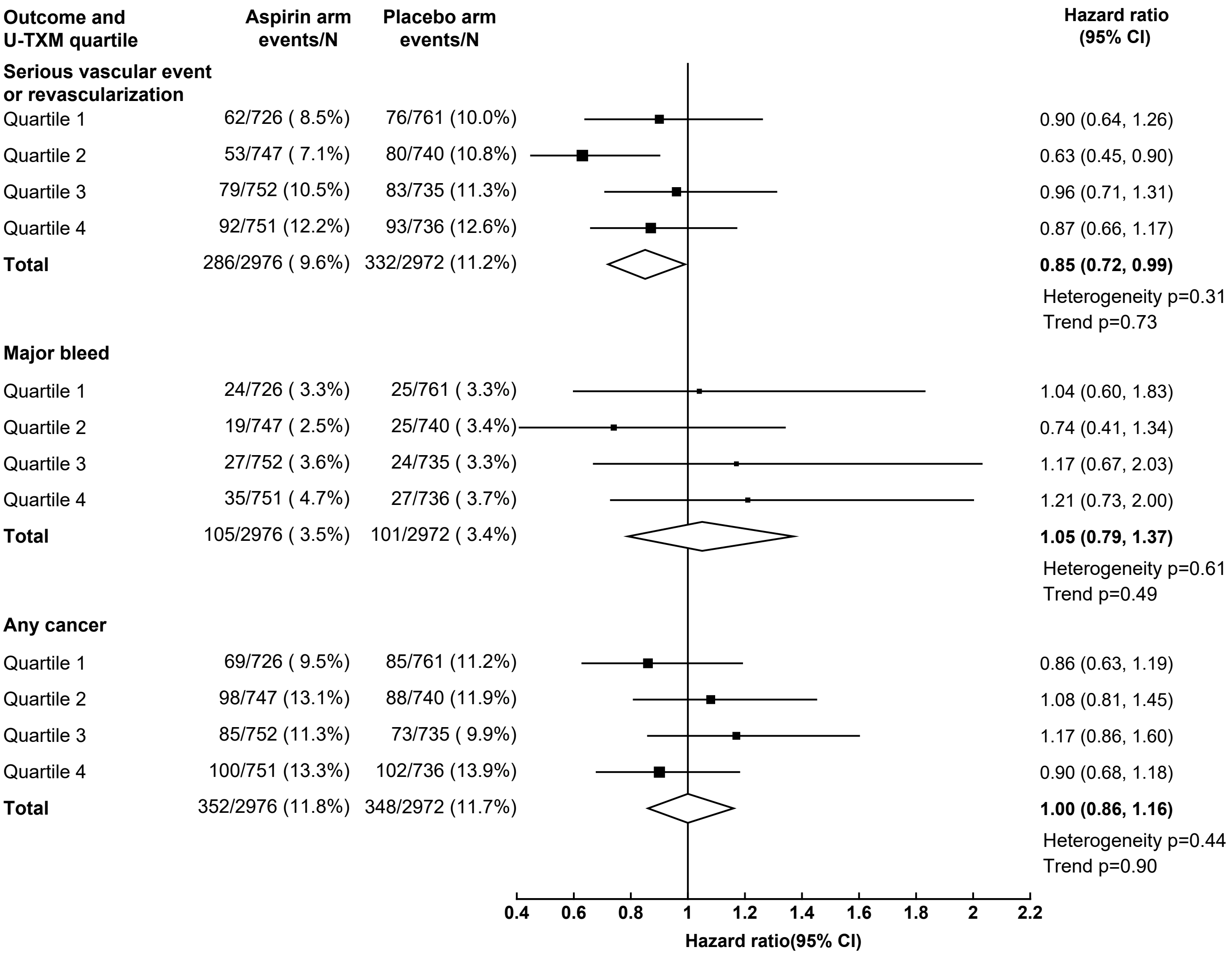
Outcome and U-TXM quartile
Aspirin arm events/N
Placebo arm events/N
Hazard ratio (95% CI)
Serious vascular event or revascularization
Quartile 1 62/726 (8.5%) 76/761 (10.0%) 0.90 (0.64, 1.26)
Quartile 2 53/747 (7.1%) 80/740 (10.8%) 0.63 (0.45, 0.90)
Quartile 3 79/752 (10.5%) 83/735 (11.3%) 0.96 (0.71, 1.31)
Quartile 4 92/751 (12.2%) 93/736 (12.6%) 0.87 (0.66, 1.17)
Total 286/2976 (9.6%) 332/2972 (11.2%) 0.85 (0.72, 0.99)
Heterogeneity p=0.31
Trend p=0.73
Major bleed
Quartile 1 24/726 (3.3%) 25/761 (3.3%) 1.04 (0.60, 1.83)
Quartile 2 19/747 (2.5%) 25/740 (3.4%) 0.74 (0.41, 1.34)
Quartile 3 27/752 (3.6%) 24/735 (3.3%) 1.17 (0.67, 2.03)
Quartile 4 35/751 (4.7%) 27/736 (3.7%) 1.21 (0.73, 2.00)
Total 105/2976 (3.5%) 101/2972 (3.4%) 1.05 (0.79, 1.37)
Heterogeneity p=0.61
Trend p=0.49
Any cancer
Quartile 1 69/726 (9.5%) 85/761 (11.2%) 0.86 (0.63, 1.19)
Quartile 2 98/747 (13.1%) 88/740 (11.9%) 1.08 (0.81, 1.45)
Quartile 3 85/752 (11.3%) 73/735 (9.9%) 1.17 (0.86, 1.60)
Quartile 4 100/751 (13.3%) 102/736 (13.9%) 0.90 (0.68, 1.18)
Total 352/2976 (11.8%) 348/2972 (11.7%) 1.00 (0.86, 1.16)
Heterogeneity p=0.44
Trend p=0.90
0.4 0.6 0.8 1 1.2 1.4 1.6 1.8 2 2.2
Hazard ratio(95% CI)

Figure S5
